# The *Burkholderia* Type VI Secretion System 5: Composition, Regulation and Role in Virulence

**DOI:** 10.3389/fmicb.2018.03339

**Published:** 2019-01-10

**Authors:** Jan Lennings, T. Eoin West, Sandra Schwarz

**Affiliations:** ^1^Interfaculty Institute of Microbiology and Infection Medicine, University of Tübingen, Tübingen, Germany; ^2^Division of Pulmonary, Critical Care and Sleep Medicine, Department of Medicine, University of Washington, Seattle, WA, United States

**Keywords:** *B. pseudomallei*, type VI secretion system, intracellular life cycle, multinucleated giant cell formation, virulence

## Abstract

The soil saprophyte and Tier I select agent *Burkholderia pseudomallei* can cause rapidly fatal infections in humans and animals. The capability of switching to an intracellular life cycle during infection appears to be a decisive trait of *B. pseudomallei* for causing disease. *B. pseudomallei* harbors multiple type VI secretion systems (T6SSs) orthologs of which are present in the surrogate organism *Burkholderia thailandensis*. Upon host cell entry and vacuolar escape into the cytoplasm, *B. pseudomallei* and *B. thailandensis* manipulate host cells by utilizing the T6SS-5 (also termed T6SS1) to form multinucleated giant cells for intercellular spread. Disruption of the T6SS-5 in *B. thailandensis* causes a drastic attenuation of virulence in wildtype but not in mice lacking the central innate immune adapter protein MyD88. This result suggests that the T6SS-5 is deployed by the bacteria to overcome innate immune responses. However, important questions in this field remain unsolved including the mechanism underlying T6SS-5 activity and its physiological role during infection. In this review, we summarize the current knowledge on the components and regulation of the T6SS-5 as well as its role in virulence in mammalian hosts.

## Introduction

*Burkholderia pseudomallei* is a soil dwelling Gram-negative bacterium that causes the potentially fatal disease melioidosis in humans and animals. Infection with *B. pseudomallei* may affect virtually any organ and may encompass a wide array of non-specific clinical manifestations ranging from acute pneumonia and sepsis to localized abscess formation, making diagnosis difficult (Currie, 2015; Wiersinga et al., 2018). The mortality rate can reach 40% despite appropriate antibiotic therapy. Southeast Asia and Northern Australia are hyperendemic regions (Cheng and Currie, 2005). However, reports of environmental *B. pseudomallei* isolates or melioidosis cases from Central and South America, Africa and South Asia indicate that the bacteria are found in the tropics worldwide (Mukhopadhyay et al., 2018; Rolim et al., 2018; Steinmetz et al., 2018; Torres et al., 2018). Furthermore, a recent comprehensive modeling study suggests vast underreporting of melioidosis cases and highlights the need to assess the true global burden and epidemiology of the disease (Limmathurotsakul et al., 2016).

*Burkholderia thailandensis* is used as a surrogate model for the Tier I Select Agent *B. pseudomallei*. Reports of human infections with *B. thailandensis* are exceedingly rare and the LD_50_ of *B. thailandensis* in mammalian animal models is at least 100 fold higher than that of *B. pseudomallei* (Smith et al., 1997; Brett et al., 1998; Lertpatanasuwan et al., 1999; Glass et al., 2006; West et al., 2008; Chang et al., 2017; Gee et al., 2018). Yet, at higher inocula via the respiratory tract, *B. thailandensis* causes rapidly fatal infections in mice and the manifestations such as neutrophil influx to the lungs, pulmonary inflammatory cytokine response, multifocal pneumonia and extra-pulmonary dissemination are similar to *B. pseudomallei* infections (West et al., 2008, 2012; Wiersinga et al., 2008a). Furthermore, both bacteria are facultative intracellular parasites and important regulatory systems and virulence factors of *B. pseudomallei* such as quorum sensing, type III and type VI secretion systems are conserved in *B. thailandensis* (Haraga et al., 2008; Majerczyk et al., 2014; Toesca et al., 2014). *B. pseudomallei* encodes six type VI secretion systems (T6SSs) and orthologs of five of them are present in *B. thailandensis* (Schell et al., 2007; Shalom et al., 2007). The analysis, so far, of three of the *Burkholderia* T6SSs revealed a high functional diversity: while the T6SS-1 and T6SS-4 are involved in interbacterial competition and metal ion acquisition, respectively, the T6SS-5 plays a central role in the intracellular life cycle of the bacteria (Schwarz et al., 2010; French et al., 2011; Russell et al., 2012; Si et al., 2017).

## The Intracellular Life Cycle of *Burkholderia Pseudomallei* and *Burkholderia Thailandensis*

Since *B. pseudomallei* is only sporadically transmitted between humans and *B. thailandensis* infections of humans are extremely rare, the capacity of the bacteria for survival and virulence in mammals likely has its origin in the exposure of the bacteria to soil dwelling predators such as protozoa (Abbink et al., 2001; Ralph et al., 2004; Fang et al., 2016). *B. pseudomallei* is able to survive phagocytosis by protozoa, which has been suggested as pre-adaptation to avoid killing by mammalian phagocytes (Gao et al., 1997; Inglis et al., 2000; Strassmann and Shu, 2017). Indeed, *B. pseudomallei* and *B. thailandensis* are able to survive inside a range of mammalian phagocytic and non-phagocytic host cells (Jones et al., 1996; Sim et al., 2009; Bast et al., 2011; Lu et al., 2012; Whiteley et al., 2017).

**FIGURE 1:**
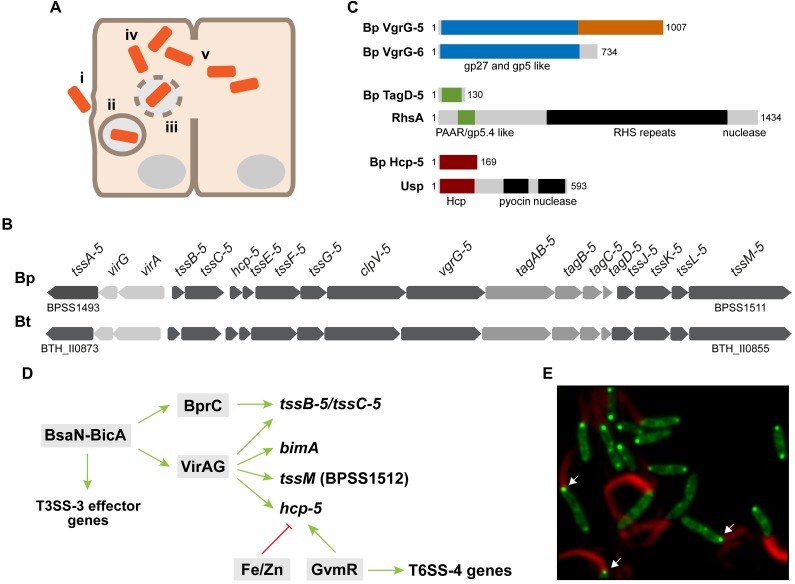
**(A)** Schematic representation of the intracellular life cycle of *Burkholderia pseudomallei* and *Burkholderia thailandensis*: (i) entry into phagocytic or non-phagocytic cell, (ii) vacuole formation, (iii) vacuolar escape before lysosomal fusion, (iv) replication and intracellular motility in cytoplasm, and (v) T6SS-5-dependent induction of plasma membrane fusion of neighboring host cell leading to MNGCs. **(B)** Genetic organization of the T6SS-5 gene cluster in *B. pseudomallei* (Bp) and *B. thailandensis* (Bt). *tss*, *tag* and regulatory genes are highlighted in dark, mid and light gray, respectively. **(C)** Schematic representation of the *B. pseudomallei* (Bp) VgrG-5, TagD-5 and Hcp-5 domain organization in comparison with Bp VgrG-6 (UniProt: Q63II0), RhsA (PAAR domain containing protein of *Dickeya dadantii;* UniProt: E0SAK8) (Koskiniemi et al., 2013) and Usp (Hcp domain containing protein of *E. coli*; UniProt: Q1RG83), respectively. Blue, green, and red indicate domains conserved in VgrG, TagD, and Hcp proteins, respectively. The VgrG-5 CTD is indicated in orange and accessory (effector) domains are highlighted in black. **(D)** Summary of T6SS-5 regulators discussed in the text that affect expression of T6SS-5 and other genes involved in host-pathogen interaction [modified from (Chen et al., 2011)]. **(E)** Epifluorescence microscopy of *B. thailandensis* expressing a chromosomal *clpV-5-sfgfp* fusion during infection of Hela cells. ClpV-5-sfGFP shows foci formation at the bacterial cell pole and a diffuse localization in the cytoplasm. Actin was stained with Texas Red-Phalloidin. White arrows indicate the occurrence of actin tail formation and T6SS-5 expression and assembly in the same bacterial cell.

A detailed discussion on the intracellular life cycle is beyond the scope of this review and we refer the reader to several comprehensive overviews on this topic (Allwood et al., 2011; Stone et al., 2014; Willcocks et al., 2016). In brief, upon passive or active entry into the host cell the bacteria are located in a membrane-bound vacuole (Jones et al., 1996; Figure 1A). Before lysosomal fusion *B. pseudomallei* and *B. thailandensis* escape the endocytic vacuole, a process that is significantly impaired in T3SS-3 mutants (Stevens et al., 2002; Vander Broek and Stevens, 2017). Once in the cytosol of the host cell the bacteria replicate and employ BimA to facilitate actin tail formation or the flagella fla2 system for intracellular motility (Stevens et al., 2005; French et al., 2011; Sitthidet et al., 2011). Intercellular spread of *B. pseudomallei* and *B. thailandensis* can occur directly without exposure of the bacteria to the extracellular milieu by the formation of multinucleated giant cells (MNGCs). MNGCs are the result of plasma membrane fusion and subsequent cytoplasmic mixing of the infected and neighboring host cell (Kespichayawattana et al., 2004; Boddey et al., 2007; French et al., 2011). These cell-cell fusions have been detected in lung tissue samples of melioidosis patients and mice infected with a low dose of *B. pseudomallei* (Wong et al., 1995; Conejero et al., 2011). Essential to MNGC formation is the *Burkholderia* T6SS-5 (also named cluster 1 T6SS) whose mechanism of action is still unknown (Pilatz et al., 2006; Burtnick et al., 2011; French et al., 2011; Suparak et al., 2011; Schwarz et al., 2014). Furthermore, findings on the T6SS-5 of *Burkholderia mallei*, which is closely related to *B. pseudomallei*, are discussed in the review (Schell et al., 2007; Losada et al., 2010).

## Components of the T6SS-5 Apparatus

The T6SS is a complex contractile injection system (CIS) exhibiting close structural and functional resemblance with other CIS such as myophage tails and R type pyocins (Veesler and Cambillau, 2011; Leiman and Shneider, 2012; Ge et al., 2015). The T6S apparatus is composed of 14 core components termed Tss (type VI secretion system) and PAAR, and variably present Tag (type VI secretion system associated) proteins serving regulatory, structural or effector functions (Shalom et al., 2007; Hsu et al., 2009; Aschtgen et al., 2010; Shneider et al., 2013; Cianfanelli et al., 2016a). Tss proteins assemble into three T6SS subcomplexes: a tubular system located in the cytoplasm consisting of the contractile sheath proteins TssB and TssC surrounding an inner tube formed by the Hcp (TssD) protein sharpened at one end by the TssI (VgrG) and PAARproteins, an envelope spanning membrane complex (TssM, TssL, and TssJ) and a base plate (TssE, TssF, TssG, and TssK) anchoring tube and sheath to the membrane complex (Cascales and Cambillau, 2012; Zoued et al., 2014; Nguyen et al., 2018). TssA was shown to initiate and coordinate sheath and tube polymerization during T6SS biogenesis (Zoued et al., 2016, 2017; Dix et al., 2018). The force-generating contraction of TssB and TssC acts as a molecular spring that pushes the inner Hcp tube tipped with the VgrG and PAAR spike proteins across the cell envelope into the target cell (Basler et al., 2012; Basler, 2015). Following translocation, the contracted sheath proteins are recycled by the ATPase ClpV (TssH) (Bonemann et al., 2009; Pietrosiuk et al., 2011; Kube et al., 2014; Kudryashev et al., 2015).

The vast majority of characterized T6SSs are employed by bacteria to inject toxic effector proteins into other prokaryotes (Russell et al., 2014a; Cianfanelli et al., 2016b; Hood et al., 2017; Sana et al., 2017). In addition, T6SSs specialized for effector protein delivery into eukaryotic cells including fungi and for the acquisition of metal ions have been described (Wang et al., 2015; Si et al., 2017; Trunk et al., 2018). This functional diversity extends to the mechanisms facilitating effector loading onto the T6SS. For instance, VgrG and PAAR (TagD) proteins can contain domains with effector function or act as carriers by binding to effector proteins while the Hcp tube can serve to translocate small (<25 kDa) effector proteins (Pukatzki et al., 2007; Silverman et al., 2013; Durand et al., 2014; Unterweger et al., 2015; Bondage et al., 2016; Ma et al., 2017; Quentin et al., 2018).

In addition to the canonical T6SS^i^ subtype described above, which is predominantly found in Proteobacteria, other pathways (T6SS^ii-iv^) have been identified that differ in composition and taxonomic distribution (Boyer et al., 2009; Broms et al., 2010; Russell et al., 2014b; Eshraghi et al., 2016; Bock et al., 2017). The *Burkholderia* T6SSs belong to the T6SS^i^ pathway. Two different nomenclatures exist for naming the T6SS gene clusters and components: cluster 1–6 T6SS (Schell et al., 2007) and T6SS-1–T6SS-6 (Shalom et al., 2007). We adopted the nomenclature T6SS-1–T6SS-6 by Shalom et al., which is also universally used to name individual T6SS proteins. The T6SS-5 [cluster 1 T6SS according to the nomenclature by Schell et al. (2007)] consists of the 13 Tss core components that are encoded by the same gene cluster (Table 1 and Figure 1B). Furthermore, four *tag* genes, *tagA/B-5*, *tagB-5*, *tagC-5* and *tagD-5*, are present in the T6SS-5 gene cluster whose role in T6SS-5 function is currently unknown. Primary sequence analysis indicates that TagA/B-5 and TagB-5 belong to the family of pentapeptide repeat proteins (PRP) (Shalom et al., 2007). Examples of characterized PRPs are the cytoplasmic quinolone resistance protein Qnr in *E. coli* and PipB2, a kinesin-recruiting T3SS effector protein in *S*. *enterica* sv. Typhimurium (Tran et al., 2005; Henry et al., 2006). TagA/B-5 is essential for MNGC formation, full virulence in mice and Hcp-5 secretion indicating a critical role in T6SS-5 activity but not as an effector protein (Hopf et al., 2014). TagD-5 is a PAAR-like protein comprising 130 amino acids that appears to lack effector domains and TagC-5 is a hypothetical protein of unknown function (DUF3540) (Figure 1C). Lastly, two regulatory genes are located within the T6SS-5 cluster encoding the two component regulator VirAG, which is required for transcriptional activation of T6SS-5 genes during infection (Chen et al., 2011).

**Table 1:** Components of the T6SS-5 gene cluster in *B. pseudomallei* and *B. thailandensis*.

Gene ID Bp^a^	Gene ID Bt^b^	Tss/Tag nomenclature	Alternative name	T6SS^i^ description/subcomplex
BPSS1493	BTH_II0873	*tssA-5*		Sheath/tube assembly coordination^c^
BPSS1494	BTH_II0872	*virG*		Two component regulator VirAG; response regulator
BPSS1495	BTH_II0871	*virA*		Two component regulator VirAG; sensor kinase
BPSS1496	BTH_II0870	*tssB-5*		Contractile sheath
BPSS1497	BTH_II0869	*tssC-5*		Contractile sheath
BPSS1498	BTH_II0868	*tssD-5*	*hcp-5*	Tail tube/needle
BPSS1499	BTH_II0867	*tssE-5*		Base plate
BPSS1500	BTH_II0866	*tssF-5*		Base plate
BPSS1501	BTH_II0865	*tssG-5*		Base plate
BPSS1502	BTH_II0864	*tssH-5*	*clpV-5*	Sheath recycling AAA^+^ ATPase
BPSS1503	BTH_II0863	*tssI-5*	*vgrG-5*	Spike protein
BPSS1504	BTH_II0862	*tagA/B-5*		Pentapeptide repeat protein
BPSS1505	BTH_II0861	*tagB-5*		Pentapeptide repeat protein
BPSS1506	BTH_II0860	*tagC-5*		Hypothetical
BPSS1507	BTH_II0859	*tagD-5*		PAAR like protein/spike tip
BPSS1508	BTH_II0858	*tssJ-5*		Membrane complex
BPSS1509	BTH_II0857	*tssK-5*		Base plate
BPSS1510	BTH_II0856	*tssL-5*		Membrane complex
BPSS1511	BTH_II0855	*tssM-5*		Membrane complex


*^a^B. pseudomallei isolate K96243; ^b^B. thailandensis isolate E264; ^c^Dix et al. (2018).*

## Regulation of T6SS-5 Gene Expression

The first evidence of the induction of T6SS-5 gene expression by a host cell derived signal has been provided by an *in vivo* expression technology (IVET) study (Shalom et al., 2007). The subsequent finding that the capability of vacuolar escape into the cytoplasm is a prerequisite for the activation of T6SS-5 genes suggested a cytoplasmic localization of the signal (Wong et al., 2015). Indeed, glutathione (GSH) and other low molecular weight (LMW) thiols such as cysteine have been identified to induce T6SS-5 gene expression (Wong et al., 2015). GSH is an antioxidant present at millimolar concentrations in the host cell cytoplasm. It contains one thiol group that acts as a reducing agent. Exposure of *B. pseudomallei* outside of host cells to reduced but not oxidized glutathione stimulated *hcp-5* expression by approximately 1000 fold (Wong et al., 2015). However, it is important to note that so far LMW thiols have been shown to induce transcription of T6SS-5 genes but not secretory activity of the T6SS-5. At present, the signal(s) necessary to elicit T6SS-5 contraction and secretion are not known.

Low molecular weight thiols are sensed by the sensor histidine kinase VirA of the two component system VirAG, which forms a dimer that is reduced by thiols to the active monomeric form (Wong et al., 2015). During infection of host cells VirAG positively regulates expression of *bimA* and T6SS-5 genes (Chen et al., 2011). *In trans* overexpression of *virAG* in *B. pseudomallei* and *B. thailandensis* activates the T6SS-5 and leads to Hcp-5 secretion in culture media (Schell et al., 2007; Burtnick et al., 2011; Schwarz et al., 2014; Toesca et al., 2014). Furthermore, the transcription of T6SS-5 and T3SS-3 genes is co-regulated by BsaN encoded in the T3SS-3 gene cluster (Figure 1D). BsaN activates T3SS-3 effector and translocon genes, *virAG* and the regulatory gene *bprC*. BprC in turn induces expression of *tssB-5* and *tssC-5* (Chen et al., 2011, 2014). Expression of T6SS-5 genes was shown to be co-regulated with that of T6SS-4 and secondary metabolite genes as well as a gene located next to the T6SS-5 gene cluster encoding a deubiquitinase that is secreted by the type II secretion system (Shanks et al., 2009; Burtnick et al., 2014; Duong et al., 2018). Lastly, transcription of T6SS-5 genes is inhibited in the presence of iron and zinc (Burtnick and Brett, 2013).

## Proteins Secreted by the T6SS-5

Taking advantage of the fact that *virAG* overexpression induces secretion of T6SS-5 in bacteria grown in culture medium, comparative mass spectrometric analysis of culture supernatants was performed to identify T6SS-5 effector proteins in *B. thailandensis*. Two proteins have been identified that were absent or of significantly lower abundance in the supernatant of a Δ*tssK-5* mutant relative to the wildtype: Hcp-5, the inner tube forming protein of T6SSs and VgrG-5, the needle spike protein (Schwarz et al., 2014). Hcp-5 does not appear to carry effector domains (Figure 1C). VgrG-5 contains an N- terminal and middle domain related to gp5 and gp27 bacteriophage spike forming proteins that are conserved in all T6SS^i^ VgrG proteins (Leiman et al., 2009). However, VgrG-5 possesses an additional domain, located at the C-terminus (VgrG-5 CTD), that is unique to the *Burkholderia* genus (Figure 1C; Burtnick et al., 2011; Schwarz et al., 2014; Toesca et al., 2014). Deletion of the VgrG-5 CTD abrogated cell-cell fusions and virulence in mice but did not affect secretion of Hcp-5 (Burtnick et al., 2011; Schwarz et al., 2014; Toesca et al., 2014). This result suggests that VgrG-5 is a specialized VgrG protein and that its CTD has essential effector function (Pukatzki et al., 2007; Durand et al., 2014; Schwarz et al., 2014). At present, VgrG-5 is the only T6SS-5 secreted protein identified with putative effector activity. Many other VgrG proteins containing additional domains at the C-terminus that display enzymatic activity, such as cross-linking of monomeric actin, have been described (Pukatzki et al., 2007; Ma et al., 2009; Suarez et al., 2010; Brooks et al., 2013). However, the VgrG-5 CTD lacks significant sequence similarity to proteins of known function and further studies will be required to determine whether the protein exhibits membrane fusion activity. Furthermore, it cannot be excluded that the protein acts as a carrier for as yet unidentified T6SS-5 effectors.

## Role of the T6SS-5 in the Intracellular Life Cycle and in Virulence *In Vivo*

Several studies established a principal role of the *B. pseudomallei* and *B. thailandensis* T6SS-5 in inducing MNGC formation (Pilatz et al., 2006; Burtnick et al., 2011; French et al., 2011; Schwarz et al., 2014; Toesca et al., 2014). The function of host cell fusion in the pathogenesis of melioidosis, however, has yet to be determined. *In vitro*, *B. pseudomallei* and *B. thailandensis* are capable of stimulating MNGC formation in a range of primary and immortalized cells (Kespichayawattana et al., 2004; Welkos et al., 2015; Whiteley et al., 2017). Obvious potential benefits of this host cell manipulation are access to nutrients provided by uninfected host cells, and localized spread and replication without exposure to extracellular immune defense mechanisms.

Like T6SS-5 mutants, T3SS-3 mutants of *B. pseudomallei* display a host cell fusion defect (Suparak et al., 2005; Muangsombut et al., 2008; Gong et al., 2011). Since T3SS-3 mutants are impaired in vacuolar escape into the cytoplasm – a requirement for the induction of T6SS-5 gene expression– the role of T3SS-3 in MNGC formation could be indirect. To clarify the function of the T3SS-3 in MNGC formation, French et al. utilized a photothermal nanoblade to place a *B. thailandensis* T3SS-3 mutant from the extracellular milieu directly into the cytoplasm of the host cell thereby bypassing endocytic vesicle enclosure and escape (French et al., 2011). The finding that the mutant was able to induce host cell fusion following nanoblade delivery conclusively demonstrated that the T3SS-3 is not involved in this process.

In addition to T6SS-5 genes and *bimA* being co-regulated by VirAG, it has been shown that the deletion of structural components of the T6SS-5 reduced actin tail formation in *B. pseudomallei* and *B. mallei* (Burtnick et al., 2010;Chen et al., 2011). The underlying basis of this effect is currently unclear. Interestingly, however, the ability of the bacteria to move in the host cell cytoplasm is a prerequisite for the stimulation of cell-cell fusion. Disruption of intracellular motility of *B. pseudomallei* and *B. thailandensis* almost entirely abolished MNGC formation (French et al., 2011). This observation suggests a site-specific induction of T6SS-5 secretion inside the host cell that leads to cell-cell fusion. Alternatively, intracellular motility was proposed to be required to bring the plasma membrane of neighboring host cells into close proximity before they are punctured by the T6SS-5 to create a hemifusion zone leading to cell-cell fusion (Toesca et al., 2014). In support of these notions, fluorescence microscopy of *B. thailandensis* expressing *clpV-5-sfgfp* during infection showed actin tail formation and T6SS-5 expression in the same bacterial cell (Figure 1E; Schwarz et al., 2014).

The deletion of essential T6SS-5 genes drastically decreased virulence of *B. pseudomallei* and *B. thailandensis* in mammalian models of acute infection (Pilatz et al., 2006; Schwarz et al., 2010, 2014; Burtnick et al., 2011; Hopf et al., 2014). Intranasal inoculation of mice with *B. pseudomallei* wildtype and *tssK-5* and *tagA/B-5* mutants showed a significant attenuation of virulence upon T6SS-5 disruption (Pilatz et al., 2006; Hopf et al., 2014). CFU measurements of lung, liver, and spleen revealed that T6SS-5 mutants were able to disseminate to distant sites although the bacterial load in the organs was significantly lower compared with wildtype challenged mice. Likewise, the LD_50_ of a *B. pseudomallei hcp*-5 mutant in hamsters after intraperitoneal challenge was 1000 fold higher than that of the wildtype (Burtnick et al., 2011). Furthermore, after high dose pulmonary infection with *B. thailandensis* wildtype all mice succumbed whereas a *tssK*-5 mutant failed to cause lethal infections and to proliferate in the lung, liver and spleen (Schwarz et al., 2010). However, the *tssK*-5 mutant caused rapidly fatal infections in mice lacking the innate immune adapter molecule MyD88, which contributes to neutrophil recruitment and activation in mice infected with *B. pseudomallei* (Wiersinga et al., 2008b). The finding that the *B. thailandensis tssK-5* mutant is highly virulent in MyD88^-/-^ mice indicates that the T6SS-5 is required to overcome MyD88-dependent immune responses to establish an infection (Schwarz et al., 2010). *In vitro*, T6SS-5 mutants are able to multiply in the host cell cytoplasm (Shalom et al., 2007). Thus, the mere ability to replicate in the intracellular compartment appears to be a necessary but not sufficient trait of *B. pseudomallei* to cause disease. Lastly, virulence of T6SS-5 mutants of *B. pseudomallei*, *B. thailandensis* and *B. mallei* was attenuated in a cockroach model of infection (Fisher et al., 2012).

## Conclusion and Future Perspective

Many fundamental questions remain unanswered since the discovery of the vital role of the T6SS-5 in *B. pseudomallei*-host cell interaction over 10 years ago. Critically, deciphering the mode of action of the T6SS-5 poses a challenge for the field as it still remains elusive although an essential candidate effector has been identified. In particular, important unsolved questions are: What is the exact subcellular localization of translocated VgrG-5 and does it function as membrane fusion protein? Is the VgrG-5 CTD sufficient for mediating cell-cell fusion or are other (T6SS-5) proteins involved in the process? Does the T6SS-5 employ host cellular factors to exert its function? In addition, investigating the molecular and cellular details of the MyD88-dependent immune response that facilitates control of T6SS-5 mutant bacteria will improve our understanding of T6SS-5 function. To answer these questions *B. thailandensis* will be an ideal model organism as work with this bacterium is less laborious and less restricted with respect to for example high throughput and *in vivo* imaging techniques compared with *B. pseudomallei*. Advancing knowledge on the molecular basis of the T6SS-5 – a key virulence determinant of *B. pseudomallei* – will benefit the development of strategies to disable the capacity of the pathogen to survive and proliferate in humans.

## Author Contributions

JL and TW wrote the manuscript. SS conceived and wrote the manuscript.

## Conflict of Interest Statement

The authors declare that the research was conducted in the absence of any commercial or financial relationships that could be construed as a potential conflict of interest.
